# Understanding the causes and consequences of measles virus persistence

**DOI:** 10.12688/f1000research.12094.1

**Published:** 2018-02-28

**Authors:** Diane E. Griffin, Wen-Hsuan W. Lin, Ashley N. Nelson

**Affiliations:** 1 W. Harry Feinstone Department of Molecular Microbiology and Immunology, Johns Hopkins Bloomberg School of Public Health, 615 N. Wolfe Street, Baltimore, MD 21205, USA; 2 Department of Pathology, Columbia University School of Medicine, New York, NY, 10032, USA

**Keywords:** measles, lymphoid tissue, RNA, viruses

## Abstract

Measles is an acute systemic viral disease with initial amplification of infection in lymphoid tissue and subsequent spread over 10–14 days to multiple organs. Failure of the innate response to control initial measles virus (MeV) replication is associated with the ability of MeV to inhibit the induction of type I interferon and interferon-stimulated antiviral genes. Rather, the innate response is characterized by the expression of proteins regulated by nuclear factor kappa B and the inflammasome. With eventual development of the adaptive response, the rash appears with immune cell infiltration into sites of virus replication to initiate the clearance of infectious virus. However, MeV RNA is cleared much more slowly than recoverable infectious virus and remains present in lymphoid tissue for at least 6 months after infection. Persistence of viral RNA and protein suggests persistent low-level replication in lymphoid tissue that may facilitate maturation of the immune response, resulting in lifelong protection from reinfection, while persistence in other tissues (for example, the nervous system) may predispose to development of late disease such as subacute sclerosing panencephalitis. Further studies are needed to identify mechanisms of viral clearance and to understand the relationship between persistence and development of lifelong immunity.

## Introduction

Measles is a highly contagious systemic viral disease that remains one of the most important causes of worldwide morbidity and mortality in children ^1^. Although progress has been made in measles control through the implementation of a two-dose strategy for delivery of the live attenuated vaccine ^2^, measles remains, or has again become, endemic in many countries ^3, 4^. Research into the pathogenesis of infection and the immune responses required for recovery from infection has been conducted primarily in macaques. Macaques develop a rash disease very similar to that of humans, and investigation of this animal model system continues to highlight interesting and important, but poorly understood, aspects of this viral infection. For instance, there is a need to define the relationship between measles virus (MeV) and the immune system, including the sites of virus replication and the mechanisms, rapidity, and effectiveness of immune-mediated virus clearance as well as the importance of persistent viral RNA when clearance is incomplete ^5, 6^.

## New insights into the pathogenesis of acute infection

**Figure 1.**
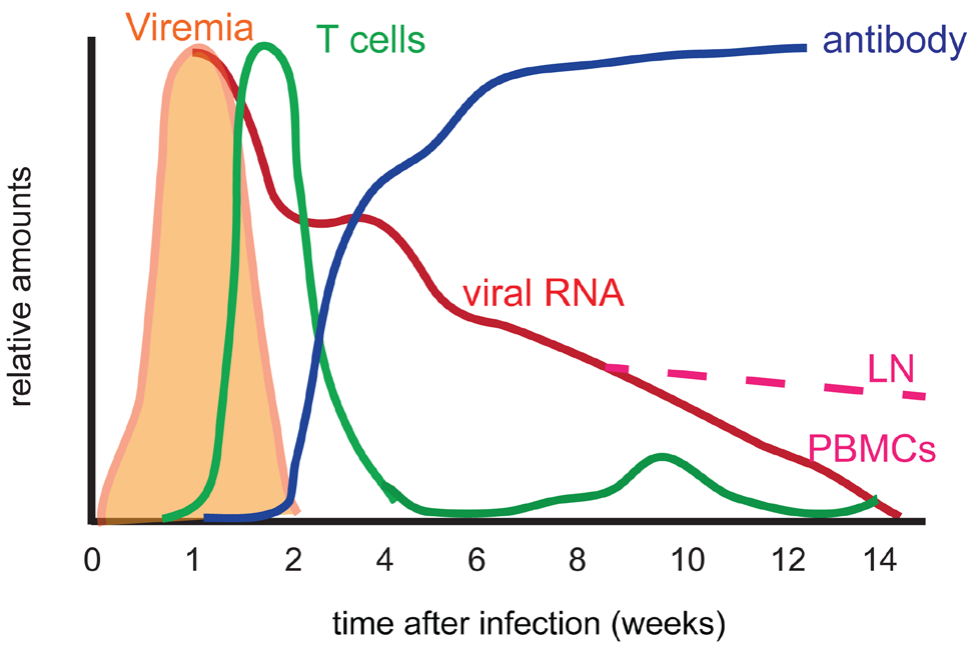
Schematic diagram of viral and immunologic features of measles during the first 3–4 months after infection. Viremia is the period during which infectious virus can be recovered from peripheral blood mononuclear cells (PBMCs). Viral RNA is cleared from PBMCs within 2–3 months but persists in lymph nodes (LNs) ^6^.

MeV, the causative agent of measles, is transmitted by aerosol or respiratory droplets and spreads from the respiratory tract to local lymphoid tissue. Studies facilitated by the development of recombinant viruses expressing reporters such as green fluorescent protein have shown that a major target for MeV replication is the immune system. Peribronchial lymphoid tissue and local draining lymph nodes initially amplify incoming virus and then export infected mononuclear cells to the blood and lymphatics for the spread of infection to more distant lymphoid tissues of the thymus, spleen, gastrointestinal tract, and peripheral lymph nodes. Virus-infected mononuclear cells in circulation also infect epithelial and endothelial cells in multiple non-lymphoid organs (for example, skin, conjunctivae, kidney, lung, and liver) ^7– 13^. This period of virus amplification and systemic spread lasts for 10–14 days after infection and is clinically silent. The onset of disease with presentation of fever and rash, a manifestation of the MeV-specific cellular immune response, coincides with the process of infectious virus clearance ^1^. Soon after the rash resolves, infectious virus can no longer be recovered, but the clearance of viral RNA occurs slowly over the next several months ^6, 14– 16^ ( Figure 1).

## Failure of the innate immune response to prevent virus dissemination

**Figure 2.**
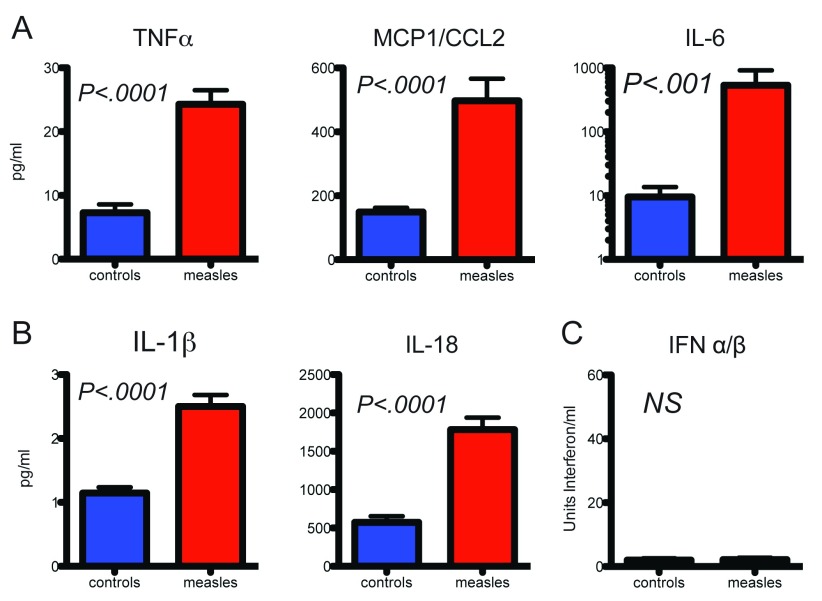
Innate immune responses to wild-type measles virus infection in humans and macaques. Measurement of levels of cytokines indicative of nuclear factor kappa B activation ( **A**) and inflammasome activation ( **B**) in plasma of children and interferon ( **C**) in monkeys at the time of rash onset ^17, 20^.

Virus amplification and spread are rapid and, in contrast with most other acute viral infections, occur without inducing signs and symptoms of acute infection or detectable amounts of type I or III interferon (IFN) ^17^ ( Figure 2). Therefore, failure of early host control of virus replication allows widespread infection. This observation raises questions as to the nature and effectiveness of the innate cellular response to MeV infection and the reason(s) for failure to induce IFN.

Cells have several mechanisms for sensing and responding to virus infection that can be initiated at the cell surface (for example, Toll-like receptors [TLRs]) or after entry and initiation of replication (for example, RNA helicases such as RIG-I and MDA5 and RNA-binding proteins such as PKR and IFIT1) ^18^. Analyses of the cytokine and chemokine profiles of children with natural measles and experimentally infected macaques early after infection suggest activation of the nuclear factor kappa B (NF-κB) (for example, interleukin-6 [IL-6], CCL2, and tumor necrosis factor alpha [TNFα]) and inflammasome (for example, NLRP3, IL-1β, and IL-18) pathways but not the IFN (for example, IFNα, IFNβ, and IFNλ) response ^19, 20^ ( Figure 2).

MeV belongs to the morbillivirus genus of the family *Paramyxoviridae*, negative-sense RNA viruses with non-segmented genomes and a lipid envelope. The envelope has surface projections composed of the viral hemagglutinin (H) and fusion (F) glycoproteins. H interacts with the virus receptor for attachment, and F interacts with H and with the cell membrane for fusion and entry. MeV targets several types of cells (for example, B and T lymphocytes, dendritic cells, monocytes, endothelial cells, and epithelial cells) and uses multiple receptors in a virus strain and cell type-specific manner, determined by the H protein ^21– 23^. Two receptors used by wild-type (WT) strains of MeV have been identified: signaling lymphocytic activation molecule (SLAM) or CD150, present on activated immune cells ^24^, and poliovirus receptor-related 4 or nectin 4, present on epithelial cells ^25– 27^. H interaction with SLAM-expressing immune cells results in preferential infection of activated cells and antigen-experienced memory T cells ^28^.

The H proteins of WT strains of MeV also interact with TLR2 ^29^, a transmembrane pathogen recognition receptor expressed on epithelial cells and most immune cells ^30, 31^. TLR2 signals through adaptor proteins MyD88 and IRAK4 to activate NF-κB, induce the transcription of mRNAs for IL-6 family member proteins, IL-1β, and TNFα, and increase the expression of SLAM ^32^. A role for TLR2 engagement during MeV infection is suggested by increases in plasma levels of soluble TLR2 as well as IL-1β, IL-6, and TNFα ^19, 20, 33^ ( Figure 2).

In contrast, MeV has evolved multiple mechanisms for inhibiting the induction of IFN, and IFN is not detectable in response to infection ^17, 34, 35^. At the cell surface, interaction with DC-SIGN on dendritic cells both facilitates MeV infection and suppresses RIG-I and MDA5 activation by preventing the PP1 phosphatase-mediated CARD domain dephosphorylation required for the induction of IFNβ ^36– 38^. In addition, after entry and during replication, the synthesis of non-structural proteins V and C encoded within the P gene occurs. The V/C/P proteins block the induction of type I IFN. The V protein binds PP1 to inhibit RIG-I and MDA5 dephosphorylation ^39^ and the helicase domain of MDA5, but not RIG-I, to inhibit MDA5 activation ^40– 42^. V indirectly inhibits the activation of RIG-I by interacting with the related RNA helicase LGP2 to induce an inhibitory association with RIG-I ^43^. In case any IFN is produced despite this blockade of induction, the V protein also inhibits IFN receptor-induced JAK-STAT signaling and the expression of antiviral IFN-stimulated genes (ISGs) by interfering with STAT2 activation ^34, 44– 46^. In addition, the N protein prevents nuclear import of activated STATs to induce the transcription of ISGs ^47^.

In general, innate responses control virus replication directly or indirectly by inducing the expression of proteins with antiviral activities such as degradation of viral RNA and inhibition of virus translation, assembly, or release ^48^. Although this has not been thoroughly investigated during measles, there is little evidence for increased expression of ISGs ^35^. Although the MeV V protein can also interfere with the activation of the NF-κB and inflammasome response pathways ^49– 51^, this appears less successful than interference with IFN induction, as there is abundant evidence that these effector products are produced during measles ( Figure 2). Therefore, the main outcome of the innate response to MeV appears to be not early control of virus replication and spread but preparation for induction of the adaptive immune response ^52^ that suppresses virus replication and clears infectious virus after dissemination has occurred.

## Adaptive immune response and measles virus clearance

After systematic MeV infection has been established, the adaptive immune response becomes the main mechanism for infectious virus control and clearance. Evidence suggests that MeV-specific T cells play a more important role than antibodies in controlling established infection. Children with agammaglobulinemia recover from measles, whereas those with impaired cellular immunity have difficulty clearing the virus, particularly from the lung and the central nervous system ^53– 56^. Consistent with these human clinical observations, depletion of CD8 ^+^ T cells from experimentally infected rhesus macaques leads to more severe disease and prolonged viremia ^57^.

The T-cell response after MeV infection in immune-competent humans and animals generally leads to efficient control of recoverable infectious MeV. However, how MeV persists in the face of an established adaptive immune response remains an open question. In general, RNA viruses use several strategies to establish persistence, which include replication in immunologically privileged sites, downregulation of virus replication and protein synthesis, and suppression of adaptive effector responses. As MeV RNA persistence can be detected in humans and macaques that are asymptomatic, replication in immunologically privileged sites is likely to be very limited. On the other hand, antibody to the MeV H protein can reduce viral protein expression on the cell surface and within cells ^58^. Antibody might then enhance viral persistence by preventing MeV-induced cell death and reducing immune elimination of infected cells.

Unlike CD8 ^+^ T cells during chronic virus infection that develop an exhausted phenotype with dampened effector functions and upregulated inhibitory receptors ^59^, exhausted or anergic MeV-specific T cells have not been identified during infection. However, several lines of evidence suggest that T-cell immunity to MeV is suppressed after the peak of clonal expansion ( Figure 1). The MeV-specific T-cell response correlates with the control of infectious virus but wanes rapidly before virus-infected cells have been eliminated. Mathematical models simulating viral replication and RNA elimination that include MeV-specific effector T cells, antibody, target cell elimination, and regulatory T-cell immunosuppression indicate that T cells alone are insufficient to eliminate viral RNA and that antibody is required ^6^.

A subsequent macaque study demonstrated that MeV RNA clearance is accelerated when the T-cell response is augmented by prior immunization that selectively primed the T-cell but not the B-cell response ^60^. The mechanism or mechanisms by which the MeV-specific T-cell response is suppressed after the initial clonal expansion remain unclear. It is possible that mechanisms involved in MeV-induced immune suppression of global T-cell responses, such as skin test responses to tuberculin, mitogen-induced proliferation, and increases in susceptibility to other infections ^61– 63^, are also affecting the maintenance and function of MeV-specific T cells. It is also possible that the dampened MeV-specific T-cell response is a consequence of selective infection and deletion of activated memory T cells, which express higher levels of the MeV receptor SLAM, as has been proposed as an explanation for impaired responses to other infections ^28^.

## Measles virus RNA persistence: is it important?

During acute infection, MeV replicates in multiple types of cells. Infectious virus is cleared within 14–18 days, but RNA can be detected in respiratory tract secretions, urine, and peripheral blood mononuclear cells for at least 3–4 months and lymph nodes for more than 6 months after infection ^6, 14, 15^ ( Figure 1). Therefore, viral RNA likely persists in both epithelial cells and cells of the immune system. However, it is not clear how long RNA persists, the specific sites of persistence, or the importance of persistence for either the virus or the host. For the virus, persistence might provide the possibility of late reactivation with transmission that would perpetuate the virus in the human population. For the host, persistence poses a risk of progressive disease or reactivation of infection, as well as the potential advantage of prolonged stimulation of the MeV-specific immune response ^5^. Recent advances in understanding the interactions of MeV with the immune system and the late consequences of infection have both improved understanding of this disease and raised new questions and controversies for further investigation.

### Chronic/late disease

Although there are reports of detection of MeV RNA from multiple tissues of normal adults ^64^, the importance of these observations is unclear. Perhaps lengthy persistence is typical and without adverse consequences. However, it is not always benign with continued replication and spread. Persistent infection of the nervous system results in the fatal disease subacute sclerosing panencephalitis in about 1 in 2,500 children who develop their primary WT MeV infection before the age of 5 ^65, 66^. Though not without controversy, MeV persistence has also been implicated in the pathogenesis of other late-appearing chronic diseases such as Paget’s disease and otosclerosis ^67– 69^.

### Maturation of the immune response

MeV-specific antibody and T cells appear with the onset of the prodrome (fever, cough, and Koplik’s spots) and rash about 10–14 days after infection. This adaptive immune response to MeV then matures over many months. MeV-specific IgM antibody can be detected during the rash, has neutralizing capacity in standard assays, and is routinely used to confirm the diagnosis of measles. There is then a shift to production of IgG, first IgG3 and then IgG1, with specificity for most structural proteins ^70, 71^. However, the IgG produced early binds MeV with low avidity and neutralizes SLAM-dependent WT virus infection of lymphocytes poorly ^72^. IgG avidity then gradually matures over several months ^73^, resulting in sustained levels of high-avidity antibody in circulation and protection from re-infection. Thus, there are progressive changes in both antibody isotype and avidity with time after recovery.

The T-cell response to infection also matures over time. The acute effector response consists of IFNγ-secreting type 1 CD4 ^+^ and CD8 ^+^ T cells and their appearance correlates with the clearance of recoverable infectious virus and a decrease in viral RNA ^6^ ( Figure 1). T cells appear in circulation in multiple waves consistent with ongoing stimulation in lymphoid tissue, but the functional capabilities change. After the initial type 1 response, T cells begin to produce type 2 and type 17 cytokines, suggesting changing stimulatory conditions in persistently infected lymphoid tissue ^74^.

## Summary

Major advances in the understanding of the pathogenesis of measles have come through detailed investigation of MeV infection in rhesus macaques using reporter viruses to identify infected tissues, reverse transcription–quantitative polymerase chain reaction to detect and quantify viral RNA in tissue, cellular assays to analyze T and B lymphocyte function, and mathematical modeling of clearance over an extended time during recovery. These studies have shown that lymphoid tissue is targeted, that MeV RNA is persistent, and that immune response maturation is ongoing for many months. A better understanding of germinal center selection of antibody-secreting cells, the process by which high-quality MeV-specific antibody develops, and the role of persistent antigen stimulation in driving T-cell and B-cell development is of interest. Identification of the cells in secondary lymphoid tissue that harbor viral RNA and protein, and likely low levels of replicating virus, for long periods of time as well as the sites of, and cells responsible for, continued viral antigen stimulation of MeV-specific B and T cells will be required for understanding the induction of lifelong immunity.
